# Insulin Receptor Isoforms Differently Regulate Cell Proliferation and Apoptosis in the Ligand-Occupied and Unoccupied State

**DOI:** 10.3390/ijms22168729

**Published:** 2021-08-13

**Authors:** Michele Massimino, Laura Sciacca, Nunziatina Laura Parrinello, Nunzio Massimo Scalisi, Antonino Belfiore, Riccardo Vigneri, Paolo Vigneri

**Affiliations:** 1 Department of Clinical and Experimental Medicine, University of Catania, 95124 Catania, Italy; vigneripaolo@gmail.com; 2 Center of Experimental Oncology and Hematology, A.O.U. Policlinico “G. Rodolico-S. Marco”, 95123 Catania, Italy; 3 Endocrinology, Department of Clinical and Experimental Medicine, University of Catania, Garibaldi-Nesima Medical Center, 95122 Catania, Italy; lsciacca@unict.it (L.S.); m.scalisi@hotmail.it (N.M.S.); antonino.belfiore@unict.it (A.B.); nosori@tin.it (R.V.); 4 Division of Hematology, Policlinico “G. Rodolico-S. Marco”, 95123 Catania, Italy; lauraparrinello@tiscali.it; 5 Institute of Crystallography, Structural Chemistry and Biosystems, CNR-ICCSB, 95100 Catania, Italy

**Keywords:** insulin receptor, insulin action, DNA damage, caspase 3, cell proliferation, apoptosis

## Abstract

The insulin receptor (IR) presents two isoforms (IR-A and IR-B) that differ for the α-subunit C-terminal. Both isoforms are expressed in all human cells albeit in different proportions, yet their functional properties-when bound or unbound to insulin-are not well characterized. From a cell model deprived of the Insulin-like Growth Factor 1 Receptor (IGF1-R) we therefore generated cells exhibiting no IR (R-shIR cells), or only human IR-A (R-shIR-A), or exclusively human IR-B (R-shIR-B) and we studied the specific effect of the two isoforms on cell proliferation and cell apoptosis. In the absence of insulin both IR-A and IR-B similarly inhibited proliferation but IR-B was 2–3 fold more effective than IR-A in reducing resistance to etoposide-induced DNA damage. In the presence of insulin, IR-A and IR-B promoted proliferation with the former significantly more effective than the latter at increasing insulin concentrations. Moreover, only insulin-bound IR-A, but not IR-B, protected cells from etoposide-induced cytotoxicity. In conclusion, IR isoforms have different effects on cell proliferation and survival. When unoccupied, IR-A, which is predominantly expressed in undifferentiated and neoplastic cells, is less effective than IR-B in protecting cells from DNA damage. In the presence of insulin, particularly when present at high levels, IR-A provides a selective growth advantage.

## 1. Introduction

The insulin receptor (IR) is a transmembrane tyrosine kinase receptor that belongs to the class of “dependence” receptors as it is not inactive when unbound to its specific ligand but rather, in this state, favors cell apoptosis by kinase-independent pathways [1,2]. Indeed, the IR influences cell survival by relying on the presence of insulin that-when bound to the receptor-induces its phosphorylation and the consequential intracellular signaling cascade, thus promoting cell proliferation and inhibiting apoptosis [3].

However, the IR is a heterogeneous protein that, via the alternative splicing of exon 11, can generate two isoforms: IR-A lacking of 36 amino-acids encoded by exon 11 and IR-B which includes those 36 amino-acidic residues at the carboxy-terminal of the alpha-subunit [4]. All human cells display IR isoforms with different relative abundance depending on cell type and stage of development. In fact, IR-A is predominantly expressed in poorly differentiated cells (e.g., fetal and cancer cells), while IR-B is predominant in mature insulin target tissues such as liver, muscle and adipose tissue [5,6].

IR-A binds with high affinity not only insulin but also Insulin-like Growth Factors 2 (IGF-2). When activated by insulin, the IR-B mediates mainly metabolic effects, whereas the IR-A, activated by either insulin or IGF-2, mediates mitogenic effects more than IR-B [5,7].

The specific activity of the two IR isoforms in the insulin-bound and unbound states is poorly understood due to the interference of the IR cognate IGF-1 receptor (IGF1-R) and the activity of hybrid IR/IGF1-R receptors [7,8,9].

To address this issue, we generated a cell model deprived of the IGF1-R expressing either no IR or only one IR isoform. These cells were employed to evaluate the effect of IR-A and IR-B on proliferation and apoptosis in the absence (unoccupied state) or presence (occupied state) of insulin.

## 2. Results

### 2.1. Characteristics of the Generated Cell Models

**Figure 1:**
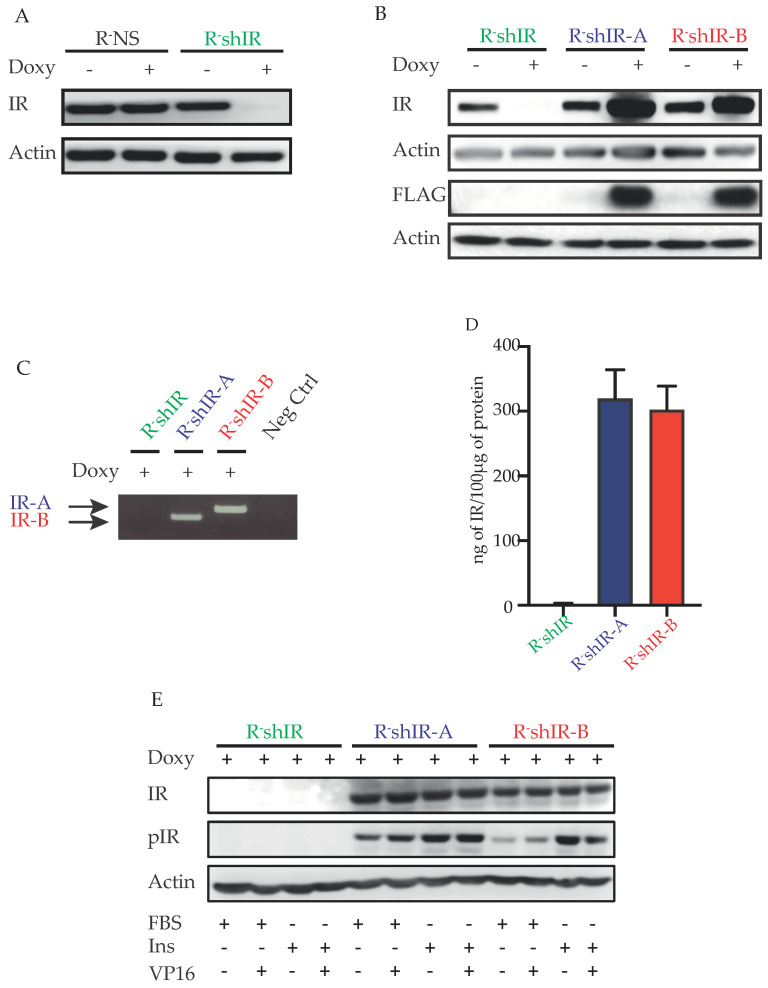
Generated cell lines: insulin receptor silencing and selective IR-A and IR-B expression. (**A**,**B**): R-NS (control cells expressing non-silencing shRNA for the IR), R-shIR (cells silenced for the IR), R-shIR-A (expressing only the human IR isoform A) and R-shIR-B (expressing only the human IR isoform B) were obtained from R-cells (cells deprived of the IGF-1 receptor and expressing only the endogenous murine IR at low levels) by transfection with the respective doxyciclyne-inducible vectors. (**C**): RT-PCR showing the selective expression of hIR-A or hIR-B in the indicated cell lines. Neg control indicates the PCR reaction missing of cDNA. (**D**): The human IRβ solid-phase sandwich ELISA was used to measure the amount of insulin receptor in each experimental condition. Histograms represent the residual endogenous receptor and the quantity of selectively expressed IR-A or IR-B in R-shIR, R-shIR-A and R-shIR-B cell lines. Bars indicate the standard deviation from two independent experiments. (**E**): Immunoblot showing the IR-A and IR-B isoforms phosphorylation in the indicate experimental conditions. Actin (Act) was used as loading control.

Anti-IR immunoblots of protein lysates derived from R-NS cells (expressing the non-silencing shRNA) and R-shIR cells (expressing the shRNA against the endogenous IR) showed that IR silencing occurred only in R-shIR cells (Figure 1A). Moreover, after exposure to doxycycline, IR silencing was followed by the selective expression of hIR-A or hIR-B in the clones lentivirally transduced with either pTRIPZ-hIRA or pTRIPZ-hIRB (Figure 1B,C). Finally, IR measurement using an ELISA-based assay documented the absence of the IR protein in R-shIR cells while R-shIR-A and R-shIR-B cells expressed similar amounts of the indicated IR isoform (Figure 1D). Furthermore, after insulin stimulation an increased phosphorylation of both IR-A and IR-B isoforms were observed (Figure 1E).

### 2.2. IR-A and IR-B Differently Affect Insulin-Dependent but Not Insulin-Independent Cell Proliferation

**Figure 2:**
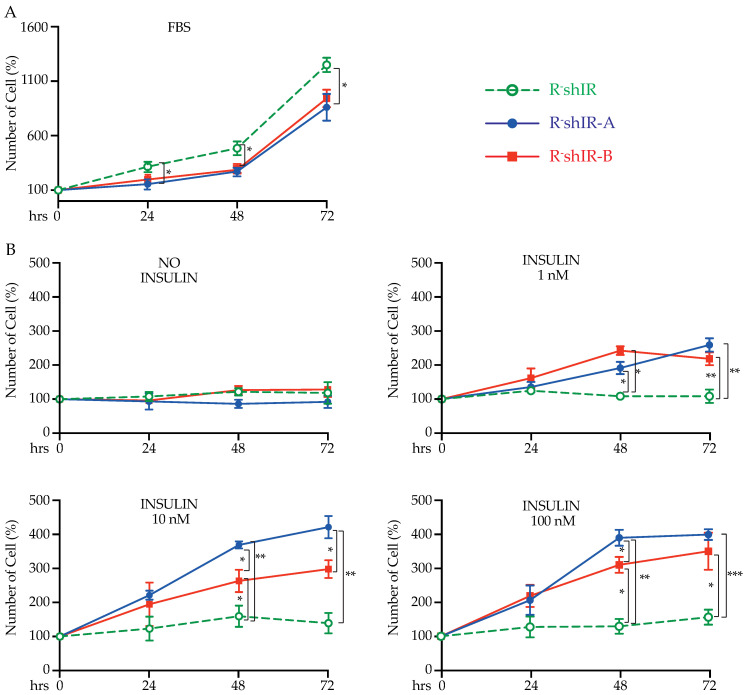
Insulin-free and insulin-occupied IR isoforms differently affect cell proliferation. In cells cultivated in the presence of doxycycline (1 µg/mL) for 72 h, viability was measured by Trypan blue exclusion. Cell number at time 0 was considered 100%. Cells were cultivated in growth medium (DMEM supplemented with 10% FBS and 2 mM glutamine) (panel **A**) or in DMEM supplemented with bovine serum albumin 0.1%, 2 mM glutamine and insulin 0, 1, 10 or 100 nM (panel **B**). Cell numbers were measured every 24 h for three days. Bars indicate the standard deviation from two independent experiments performed in triplicates. Statistical analysis of variance (ANOVA) plus Bonferroni’s post-test were calculated using the GraphPad Prism software version 8.0. (* *p* < 0.05, ** *p* < 0.01, *** *p* < 0.001).

In order to evaluate whether the unoccupied (i.e., in the absence of insulin) IR-A and IR-B differently affected cell proliferation, we expanded each cell line in medium containing 10% FBS. R-shIR cells (not expressing the IR) displayed a significantly higher growth rate relative to both R-shIR-A and R-shIR-B, an effect that was evident after 24 h and was maintained for up to 72 h for both R-shIR-A and R-shIR-B cells (two-way ANOVA plus Bonferroni post-test, *p* < 0.05) (Figure 2A). No difference was detected between the two cell types. These data indicate that both IR-A and IR-B, when unoccupied, similarly inhibit cell proliferation.

We next evaluated cell proliferation in the presence of insulin. In these experiments, cell numbers were measured before and after exposure to insulin 0, 1, 10 or 100 nM for 24, 48 and 72 h (Figure 2B). At low insulin concentrations (1 nM), R-shIR-A and R-shIR-B cells displayed a significantly higher proliferation rate than R-shIR with no major difference between the two cell lines (Figure 2B). However, after a 48 or 72-h exposure to higher insulin concentrations (10 and 100 nM), cell proliferation increased significantly more in R-shIR-A than in R-shIR-B cells (two-way ANOVA plus Bonferroni post-test) (Figure 2B). As expected, the presence or absence of insulin had no effect on R-shIR cells’ proliferation.

### 2.3. IR-A and IR-B Differently Affect Apoptosis in the Presence and Absence of Insulin

**Figure 3:**
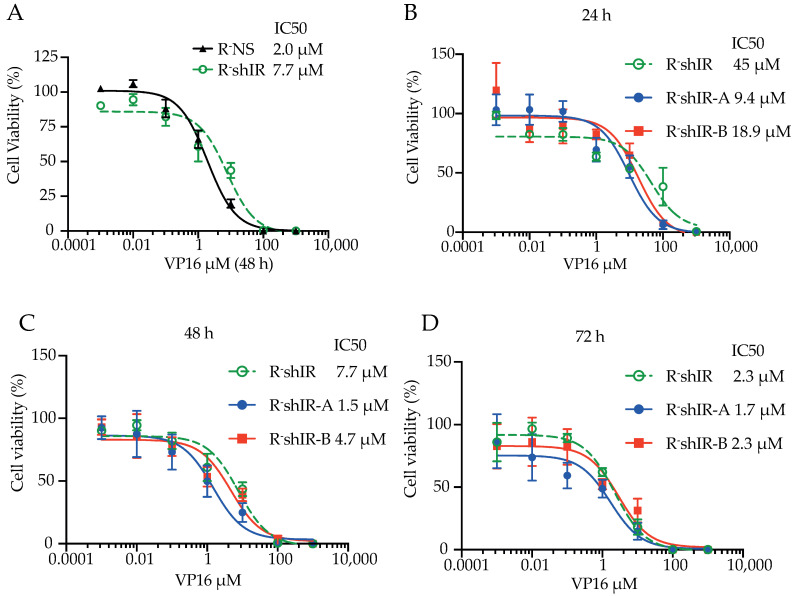
Insulin-unbound IR-A and IR-B differently affect cell viability after VP-16-induced DNA double-strand break. (**A**–**D**): The indicate cell lines, cultivated in DMEM supplemented with 10% FBS and 2 mM glutamine, were exposed to increasing logarithmic concentrations of etoposide (VP-16). Cell viability was measured with the MTS assay and data expressed setting at 100% the OD450 nm obtained from untreated cells. VP-16 half-maximal inhibitory concentration for cell viability (IC50) was obtained by logistic non-linear regression using the GraphPad Prism software version 8.

The effect of the IR isoforms on cell death due to VP-16-mediated DNA double-strand breaks was first examined in the absence of insulin (unoccupied IRs). Cells were exposed to increasing logarithmic concentrations of VP-16 for 48 h in growth medium containing 10% FBS and viability was measured using the MTS assay. R-shIR cells required higher VP-16 concentrations than R-NS cells (expressing the endogenous murine IR) to achieve the IC50 value (IC50 7.7 µM for R-shIR and 2.0 µM for R-NS, *p* < 0.001) (Figure 3A). Hence, absence of IR signaling caused resistance to VP-16-induced cytotoxicity. 

In parallel experiments unoccupied IR-A or IR-B expressing cells responded differently to DNA double-strand break (Figure 3B–D). As demonstrated by the lower IC50 values, both R-shIR-A and R-shIR-B cells reduced cell resistance to VP-16 cytotoxicity compared to R-shIR. However, at all times, IC50 values were lower in R-shIR-A cells than in R-shIR-B cells, indicating that the unoccupied IR-A is less proficient than IR-B in protecting from DNA double-strand breaks.

**Figure 4:**
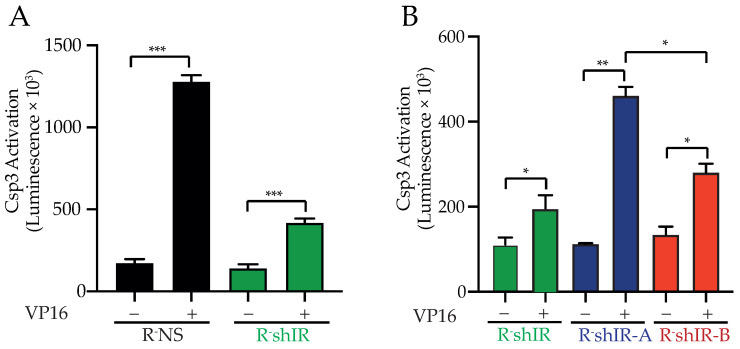
Insulin-unbound IR-A and IR-B differently affect the Caspase 3 activation after VP-16-induced DNA double-strand break. In (**A**) Caspase-3 activation was measured before and after exposure of the indicated cells to VP-16 7.7 µM (IC50 R-shIR) for 48 h, while in (**B**) cells were exposed to VP-16 45 µM (IC50 R-shIR) for 24 h (See Figure 3). Histograms indicate the luminescence value of cleaved caspase-3 and bars indicate the standard deviation obtained from three independent experiments. GraphPad Prism software version 8.0 was used to perform two-tailed t-test with 95% of confidence intervals (* *p* < 0.05, ** *p* < 0.01, *** *p* < 0.001) between two specific groups.

These observations were confirmed by measuring the activation of caspase-3, the major executioner of apoptosis. After exposure to VP-16, caspase-3 activation in R-shIR cells was reduced (untreated vs. VP-16 treated = 3 fold) relative to R-NS cells (untreated vs. VP-16 treated = 7.5 fold) (*p* < 0.001) (Figure 4A).

In agreement with these observations, after exposure to VP-16 (45 µM) caspase-3 activation was significantly greater in R-shIR-A cells (4.1 fold) compared to R-shIR-B (2.08 fold, *p* < 0.05) (Figure 4B) indicating that the unoccupied IR-A is less effective in protecting cells from VP-16 toxicity than the unoccupied IR-B.

**Figure 5:**
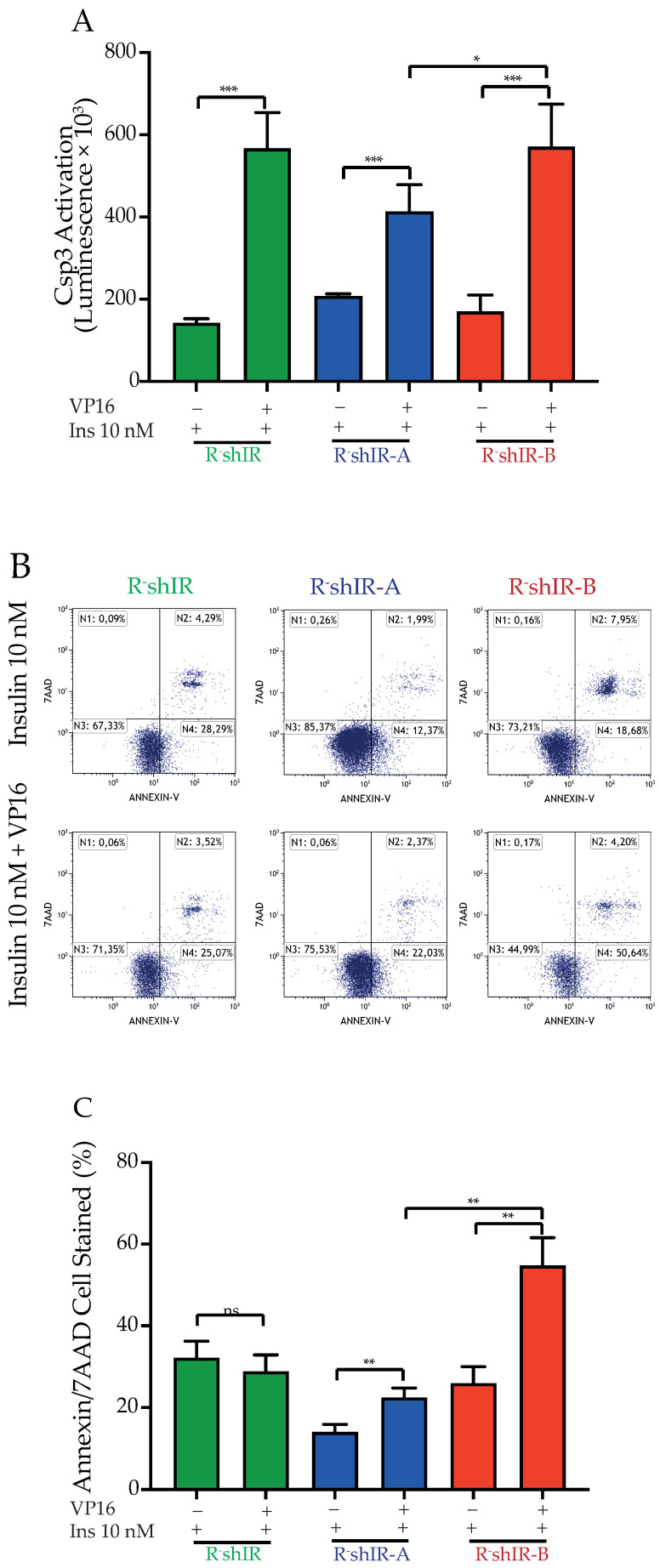
Insulin-occupied IR-A reduces caspase-3 activation and protects cells from apoptosis after DNA double-strand break. R-shIR, R-shIR-A and R-shIR-B cells, exposed to doxycycline 1 µg/mL for 72 h, were grown in DMEM supplemented with bovine serum albumin 0.1% and 2 mM glutamine and exposed or not to VP-16 in the presence of 10 nM insulin for 24 h. (**A**): caspase-3 activation; histograms represent the average of the luminescence value of cleaved caspase-3 and the bars indicate standard deviation obtained from three independent experiments. (**B**): Cell staining (Annexin A5 FITC/7-AAD, Beckman Coulter Kit) was performed with cytofluorometric analysis employing Cytomics FC500. (**C**): Average values of Annexin V and 7-AAD-positive cells after exposure to only insulin or in the presence of both insulin and VP-16. Bars indicate the standard deviation from two independent experiments. The GraphPad Prism software was used to perform two-tailed t-test with 95% of confidence intervals (* *p* < 0.05, ** *p* < 0.01, *** *p* < 0.001).

To evaluate the effect of insulin-bound IR isoforms on the apoptotic response to DNA damage, we first measured caspase-3 activation in R-shIR, R-shIR-A and R-shIR-B cells exposed to VP-16 in the presence of 10 nM insulin. A significant (*p* < 0.001) increase in caspase-3 activity was observed in all cases but this increase was significantly lower (*p* < 0.05) in R-shIR-A than in R-shIR-B and R-shIR cells (Figure 5A) suggesting an increased capacity of insulin-bound IR-A, relative to IR-B, to protect from VP-16 cytotoxicity. The finding that, after exposure to VP-16, caspase-3 activation is similar in R-shIR and R-shIR-B cells in spite of a much greater death rate in R-shIR-B cells (Figure 5B) may reflect different caspase-independent cell death processes between the two cell lines. Indeed, different levels of anti-apoptotic factors such as the inhibitor of apoptotis proteins (IAP) that bind and inactivate caspases has been described in cells deprived of the IR and the IGF1-R [1].

The difference between IR-A and IR-B was also confirmed by flowcytometry (Figure 5B,C). In the presence of insulin, R-shIR-A cells were more protected from VP-16 (cell death increased by 1.5 fold) than R-shIR-B (2.2 fold, *p* < 0.01). Finally, the difference in the death rate between R-shIR-B andR-shIR-A was 2.5 fold (*p* < 0.01, Figure 5C). As expected, no effect of insulin was seen in R-shIR cells (Figure 5B,C).

## 3. Discussion

This study provides novel insight on the biology of unbound or insulin-bound IR variants using an IGF1-R-deprived cell model expressing no IR or, selectively, the IR-A or IR-B isoforms.

In agreement with what was previously reported, in our model cells that do not express the IR were more resistant to apoptosis, confirming that unbound IRs produce pro-apoptotic signals [1,10].

IR-A and IR-B isoforms may elicit partially different intracellular signaling and biological effects upon binding to different ligands. It has been reported that in cells lacking the IGF1-R and expressing only the IR-A, insulin induces a more prolonged ERK activation than in IR-B isoform expressing cells [11]. Moreover, when IR-A is activated by IGF-2 it stimulates the p70S6 kinase at a higher level than insulin [7]. Finally, IR-A seems to predominantly mediate mitogenic effects while IR-B predominantly mediates metabolic effects.

Both IR-A and IR-B, albeit to a different extent, can reverse the effects of IR absence on cell proliferation and apoptosis. Indeed, in the absence of insulin, R-shIR-A and R-shIR-B reduce cell growth relative to R-shIR cells. On the contrary, when limited insulin is present, both IR-A and IR-B favor cell proliferation. However, at high insulin concentrations (10 nM and 100 nM) R-shIR-A cells grow significantly more than R-shIR-B cells. The difference between the two isoforms is more pronounced in regulating apoptosis. In the unoccupied state, IR-B is 2–3 fold more effective than IR-A in reducing cell resistance to DNA damage. This difference is fully reversed when insulin is added to the culture media as insulin-bound IR-A confers a much higher resistance to the cytotoxic action of VP-16 than IR-B.

In summary, both IR isoforms reduce cell growth when unbound to insulin and promote cell growth in the presence of their ligand; the latter effect is greater for IR-A. Moreover, in the unoccupied state both isoforms reduce cell resistance to DNA damage relative to cells not expressing the IR, with IR-B more effective than IR-A. In contrast, after insulin binding, IR-A becomes 2.5 fold more effective than IR-B in protecting cells from apoptosis.

Therefore, we conclude that in physiological in vivo conditions in which insulin is present, IR-A is more effective than IR-B in stimulating proliferation and protecting from apoptosis, both characteristics of pro-carcinogenic factors. Considering that IR-A is also a high affinity receptor for IGF-2 [5,12], this isoform displays oncogenic properties providing a selective growth advantage to cancer cells [13,14,15,16]. In contrast, in the presence of insulin, IR-B may have anti-cancer effects limiting cell proliferation when overexpressed [17]. This may be an intrinsic characteristic of IR-B signaling but also the result of ligand sequestration in competition with IR-A. In cancer cells that predominantly express IR-A, cell cycle promotion and escape from apoptosis can contribute to the increased cancer incidence and mortality observed in hyper-insulinemic diabetic and obese patients [18,19]. Different expression of the IR-A and IR-B isoforms and their ratio may alter the balance between mitogenic and metabolic effects of insulin in tissues. The predominant IR-B expression in liver might have a protective role against the mitogenic effects of the high concentrations of insulin in this tissue for the “first pass” of insulin secreted by the pancreas. On the contrary, elevated levels of IR-A and a high IR-A:IR-B ratio, typical of cancer cells, could contribute to an increased resistance to apoptosis in cancer patients with hyperinsulinemia, typical of all conditions of insulin resistance such as obesity and type 2 diabetes. In these patients strategies to selectively block IR-A mediated insulin action could reduce cell proliferation with beneficial effects on cancer progression [7].

In addition to cell proliferation and survival the differences between the two IR isoforms may include additional functions such as metabolism regulation and differentiation. However, the murine fibroblast model employed in this study is unsuitable to study these functions that will require further investigations.

## 4. Materials and Methods

### 4.1. IGF-1 Receptor Deprived Cells

R−mouse embryo fibroblasts (3T3-like mouse cells derived from animals with a targeted disruption of the IGF1-R gene) expressing endogenous IR at approximately 5 × 10^3^ copies per cell [8,9,11] were kindly provided by Dr. R. Baserga (Kimmel Cancer Center, Jefferson University, Philadelphia, PA, USA). R−cells were cultivated as previously reported [5].

### 4.2. Generation and Transduction of Lentiviral Vectors

pGIPZ lentiviral vectors constitutively expressing an shRNA against the murine insulin receptor (mIR) (shRNA-mIR, CLONE ID: V2LMM_176881, V2LMM_176884, V2LMM_75164, V2LMM_79169, V3LMM_438244, V3LMM_452879) or the non-silencing (NS) control (RHS4346) were purchased from Dharmacon. Doxyciclyne-inducible pTRIPZ lentiviral vectors expressing shRNA-mIR (pTRIPZ-shRNA-mIRs) and shRNA-NS (pTRIPZ-shRNA-NS) were obtained following the manufacturer’s protocol (Dharmacon).

Doxyciclyne-inducible pTRIPZ-hIRA-FLAG and pTRIPZ-hIRB-FLAG lentiviral vectors were cloned from RG215257-hIR-A or RG215691-hIR-B plasmids (all from Origene) employing the following primers containing the FLAG sequence in the reverse primer:

Fw -hIRA and -hIRB:

5′-CGCACCGGTGCCACCATGGCCACCGGGGGCCG-3′;

Rv -hIRA and -hIRB:

5′-CGACGCGTCCTAGGTAATACGACTCACTATAGGGTTACTTATCGT

CGTCATCCTTGTAATCGGAAGGATTGGACCGAGGC-3′.

The obtained cDNAs were ligated in pTRIPZ vectors using the Age-I and Mlu-I restriction sites. Recombinant lentiviruses were produced, concentrated and used for transduction as previously described [20].

### 4.3. Generation of R-NS, R-shIR, R-shIR-A and R-shIR-B Cell Lines and IR Measurement

R-cells were transduced with a mixture of each pTRIPZ-shRNA-mIRs or pTRIPZ-shRNA-NS and then exposed to 3.5 µg/mL puromycin to select resistant clones obtaining R-shIR cells (silenced for the endogenous IR) and R-NS (not silenced for the endogenous IR). To generate R-shIR selectively expressing pTRIPZ-hIRA-FLAG or pTRIPZ-hIRB-FLAG, a single R-shIR clone showing efficient murine insulin receptor silencing was obtained using a clonogenic growth assay. Protein lysates derived from each colony were subjected to an anti-IR immunoblot. The colony showing major mIR gene silencing (R-shIR) was then lentivirally transduced with pTRIPZ-hIRA-FLAG or pTRIPZ-hIRB-FLAG. Selected colonies displaying comparable amounts of human IR-A (hIRA) or human IRB (hIRB) were obtained by clonogenic growth assays, using an anti-FLAG antibody. For all experiments cells were cultivated in the presence of doxycycline (1 µg/mL) for 72 h before to perform the experiments. For IR-A and IR-B protein quantification cells were lysed in RIPA buffer and the total protein amount quantify by Pierce BCA protein assay kit (Thermofisher, Waltham, MA, USA). Each lysate was then subjected to human IRβ solid-phase sandwich ELISA (Thermofisher) to quantify the total IR-A and IR-B protein content.

### 4.4. Immunoblot

For immunoblot analysis cell lysates were separated by SDS-PAGE, transferred on nitrocellulose membranes and then hybridized with the following antibodies: anti-insulin receptor (IR) (Santa Cruz) (dilution 1:1000), anti-phosphoIR (Cell Signaling) (dilution 1:1000), anti-actin (loading control) (dilution 1:10,000) and anti-FLAG (dilution 1:1000) (both from Sigma). Appropriate horseradish peroxidase-conjugated secondary antibodies (dilution 1:1000) (Amersham Bioscience, Amersham, UK) were used to detect the indicated proteins using the LiteAblot enhanced chemiluminescence reagent (EuroClone, Milan, Italy). Signals were acquired with the C-DIGIT instrument (Licor, Lincoln, NE, USA).

### 4.5. RNA Extraction and RT-PCR

Total RNA was extracted using the Trizol reagent (Invitrogen, Waltham, MA, USA). 1 μg of RNA were reverse transcribed with Moloney murine leukemia virus reverse transcriptase and random hexamers (both from Invitrogen). Synthetized cDNA was used for PCR amplifications employing Taq DNA polymerase (Invitrogen) using the following primers: IR Fw 5′-CCAAAGACAGACTCTCAGAT-3′, IR Rv 5′-AACATCGCCAAGGGACCTGC -3′.

### 4.6. Cell Proliferation, Caspase 3 Activation and Apoptosis Measurements

Cell proliferation was assessed using the trypan blue exclusion assay and counting the cell number for three days.

The etoposide (VP-16) half-maximal inhibitory concentration (IC50) was calculated measuring cell viability every 24 h employing the MTS CellTiter 96^®^ Aqueous One Solution Cell Proliferation Assay (Promega, Madison, WI, USA). IC50 values were obtained by logistic non-linear regression.

For Caspase-3 activity, an equal amount of cell lysate was obtained from 1 × 10^6^ of cells exposed or not to VP-16. The enzyme activity was detected measuring the luminescence emission by caspase-3 cleavage using EnzCheck assay according to manufacturer’s protocol (Thermofisher Scientific, Waltham, MA, USA).

For FACS analysis, cells cultivated in the presence of 10 nM insulin were either left untreated or exposed to VP-16 (45 µM for 24 h). Cells were then washed in could PBS and double stained with Annexin V FITC/7AAD (Beckman Coulter, Brea, CA, USA) as previously reported [21].

### 4.7. Statistical Analyses

All data were expressed as mean ± standard deviation. Prism Software version 8.0 (GraphPad Software, San Diego, CA, USA) was used to perform unpaired two-tailed t-test with 95% confidence or analysis of variance (two-way ANOVA) plus Bonferroni’s post-test. A two-sided *p* < 0.05 was considered significant.

## Data Availability

Not applicable.
